# Biomechanical Modelling of Porcine Kidney

**DOI:** 10.3390/bioengineering11060537

**Published:** 2024-05-24

**Authors:** Aadarsh Mishra, Robin O. Cleveland

**Affiliations:** Department of Engineering Science, University of Oxford, Wellington Square, Oxford OX1 2JD, UK; aadarsh.mishra@spc.ox.ac.uk

**Keywords:** viscoelasticity, rheology, porcine kidney, lower pole, middle pole, upper pole, fractional model, frequency test, relaxation test, strain test

## Abstract

In this study, the viscoelastic properties of porcine kidney in the upper, middle and lower poles were investigated using oscillatory shear tests. The viscoelastic properties were extracted in the form of the storage modulus and loss modulus in the frequency and time domain. Measurements were taken as a function of frequency from 0.1 Hz to 6.5 Hz at a shear strain amplitude of 0.01 and as function of strain amplitude from 0.001 to 0.1 at a frequency of 1 Hz. Measurements were also taken in the time domain in response to a step shear strain. Both the frequency and time domain data were fitted to a conventional Standard Linear Solid (SLS) model and a semi-fractional Kelvin–Voigt (SFKV) model with a comparable number of parameters. The SFKV model fitted the frequency and time domain data with a correlation coefficient of 0.99. Although the SLS model well fitted the time domain data and the storage modulus data in the frequency domain, it was not able to capture the variation in loss modulus with frequency with a correlation coefficient of 0.53. A five parameter Maxwell–Wiechert model was able to capture the frequency dependence in storage modulus and loss modulus better than the SLS model with a correlation of 0.85.

## 1. Introduction

Soft tissues such as kidneys exhibit a stress–strain response that is viscoelastic, i.e., the mechanical properties are dependent on the strain rate. Accurate and reliable constitutive models that can accurately predict the stress–strain behaviour of soft tissues are useful for developing numerical simulations of the tissue response to mechanical loads in physiological conditions, trauma conditions and medical interventions [1]. Access to human tissue is limited and complex; however, porcine kidneys are anatomical and physiological to human kidney and so are commonly used for biomedical research [1]. Here, the shear modulus at three different locations in porcine kidneys was measured and used to determine the parameters of two constitutive models.

The primary motivation for this work is understanding the response of kidney tissue to the mechanical load created either in shock wave lithotripsy (SWL) [2,3] or burst wave lithotripsy (BWL) [4]. These are two non-invasive clinical methods for treating kidney stones, where acoustic waves are focused onto stones in order to fragment them into small enough pieces that can be passed naturally. In SWL, repetitive short-duration (~2 µs) shock waves typically fired at 1–2 Hz have been shown to result in damage to blood vessels and tubules in both clinical reports and experiments with pigs [2]. It has been proposed that a cumulative build-up of shear may be responsible for the injury [5]. In BWL, tone bursts with a centre frequency of 170 kHz or 335 kHz are fired at 10–40 Hz and are used to fragment stones. A study [6] with pig kidneys suggested that about half the BWL-treated sites suffered injury (as determined by Magnetic Resonance Imaging and histomorphometry), which was localised to kidney’s upper, middle and lower poles. Our long-term goal is to be able to predict the tissue strain that can develop in kidney tissue during either SWL or BWL; this requires a constitutive model and an investigation as to whether different regions of the kidney have different properties.

Commonly used techniques in biomechanical modelling are finite element analysis (FEA) and experimental validation. The mechanical behavior of renal tissues under varied loading conditions can be simulated using finite element analysis (FEA) [7,8,9,10,11]. Developing a constitutive model for the kidney is another example where biomechanical modelling can be used to gain insight into either an injury process or a medical procedure. For example, stones that are too large to be treated by non-invasive or ureteroscopic procedures can be treated percutaneously by placing creating a sheath access from the skin to the kidney through which tools can be inserted [12]. Biomechanical modelling can also lead to designing tools that minimise the loading and tissue damage [13,14]. Biomechanical models are also employed in automotive safety research to understand damage to organs in impact [15,16,17], sports injuries [18] and elastography methods [19,20,21]. The key parameters considered in the biomechanical modelling of porcine kidney in our study were mechanical properties and tissue deformation. Mechanical properties consist of parameters such as the shear storage modulus and loss modulus and the changes in these parameters were assessed as a response to frequency and time. Tissue deformation was investigated by applying mechanical loads such as torsion and obtaining the stress–strain response of kidney samples.

Most constitutive models to date have represented the kidney as a small number of springs and dashpots such as the Kelvin–Voigt model, Maxwell model, Standard Linear Solid (SLS) model and Maxwell–Wiechert model [22,23,24,25]. The limitations of the Kelvin–Voigt model include its assumption of a constant storage modulus (i.e., stiffness) over frequency, which has not been observed in soft biological matter, and that it cannot capture stress relaxation. Limitations of the Maxwell model include a prediction of decreasing loss modulus with frequency, which is not observed [26,27], and that it cannot capture strain creep [28,29,30,31,32]. The SLS model uses two springs and a dashpot and can capture both creep and stress relaxation response of soft tissues [28,33], but the model cannot capture soft tissues with multiple time scales or power-law properties. The Maxwell–Wiechert model uses a series of parallel elements to capture multiple time scales [22,23,24,25] as are often observed in biological tissues and has been used to capture a variety of tissues including tendons [34], breast [35] and skin [36].

An alternative constitutive framework to capture the frequency dependence of soft tissue is to use fractional viscoelastic models that employ elements with fractional derivatives that effectively operate as convolutions in the time domain and inherently have viscous and elastic properties [37]. Fractional or springpot elements capture the power law dependence that is often observed in soft tissue [38]. Fractional viscoelastic models have been applied to biological materials such as brain [23,39], epithelial cells [40,41], breast tissue cells [40,42,43], lung parenchyma [40,44] and red blood cell membranes [40,45].

There are several studies that have also investigated the mechanical properties of porcine kidney. Mechanical testing on kidneys in uniaxial compression [46] reported that the average rupture stress in the radial direction of porcine kidney cortex tissue amounted to about 0.25 MPa; the corresponding rupture strain was ~50% and the average rupture stress in the tangential direction was about 0.18 MPa with the same rupture strain of ~50%. A nominal stress of ~0.14 MPa and a nominal strain of ~30% was obtained from the uniaxial tensile tests on the cortex tissue [46]. Shear punching tests were also performed and the punching shear stress varied from 0.025 MPa to 0.035 MPa [46]. Oscillatory shear tests were performed on porcine kidney [1], which suggested a linear viscoelastic strain limit of 0.2% and a shear storage modulus of ~2.5 kPa.

In this paper, the viscoelastic mechanical behaviour of porcine kidney was characterised using dynamic testing to determine the storage and loss moduli as a function of frequency, and a step shear measurement in the time domain. One novel aspect of the work is measurements were taken for samples taken from the lower pole, middle pole and upper pole regions of the kidney. The frequency and time-domain data were fitted to a SLS model and fractional derivative model and the properties associated with the models were compared. This paper is based on work reported in the lead author’s doctoral thesis [47].

## 2. Materials and Methods

### 2.1. Preparation of Samples

Porcine kidneys of eleven Yorkshire female pigs weighing between 45 kg and 50 kg were acquired from a local butcher and the samples were stored in University of Wisconsin (MP-UW) solution (Machine Perfusion Solution-Belzer UW; Bridge to Life (Europe) Ltd., London, UK) at 4 °C. The UW solution effectively preserves kidneys for 48–72 h [48,49]. All mechanical tests were performed within 3–4 h of collection and 24 h after slaughter.

**Figure 1:**
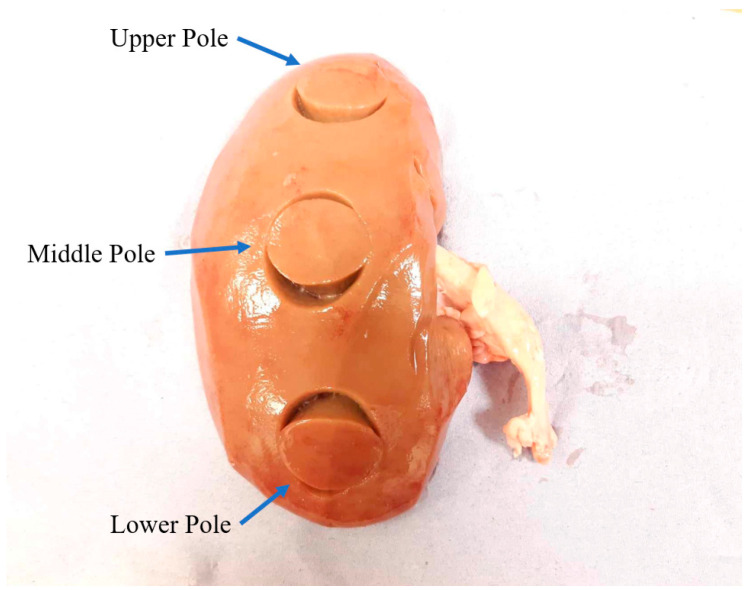
Upper pole, middle pole and lower pole of porcine kidney tested in the study.

**Figure 2:**
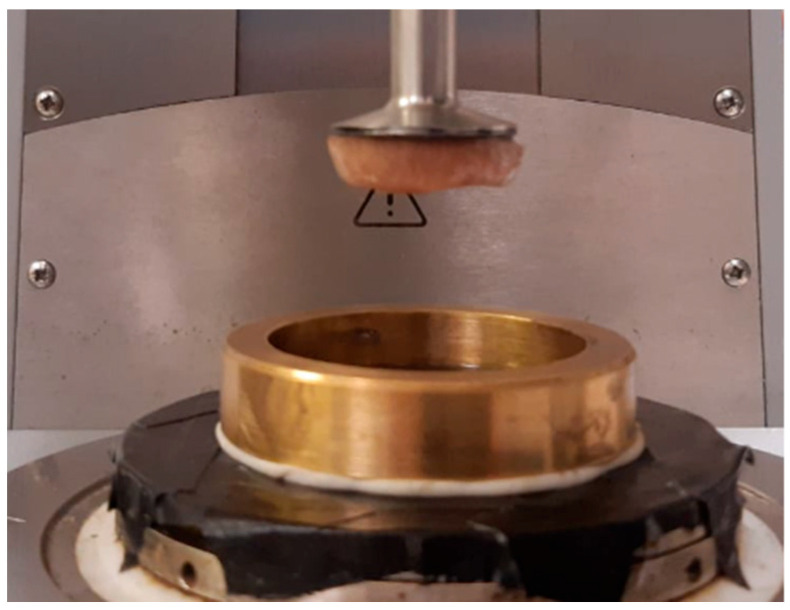
Top and bottom plate of stress-controlled rheometer (Physica MCR 301) along with the metallic container and kidney tissue attached to the upper plate.

Samples were extracted from the upper pole, middle pole and lower pole of the porcine kidney using a 25-mm diameter cork borer, see Figure 1. Samples were cut to 5–6 mm thick slices using a surgical blade. A Physica MCR 301 stress-controlled rheometer was used to measure the properties of the sample. It is a two-plate device in which the lower plate is fixed while the top plate applies torque to the sample, see Figure 2. The torque applied and the angle of deformation are measured by the rheometer, which translates these into shear stress and strain, and then calculates the storage modulus G′ and loss modulus G″. For the data here, the sample was compressed axially by 0.1 N load. To minimize slippage at the sample-plate interface, 200-grit sandpaper was affixed to the rheometer’s top and lower plates. A specially designed metallic container was fastened to the rheometer’s bottom plate in order to hydrate the samples with Phosphate Buffered Saline (PBS) solution during tests in order to maintain pH and osmolarity of the sample. The temperature of metallic container was kept at 37 °C.

### 2.2. Viscoelasticity

**Figure 3:**
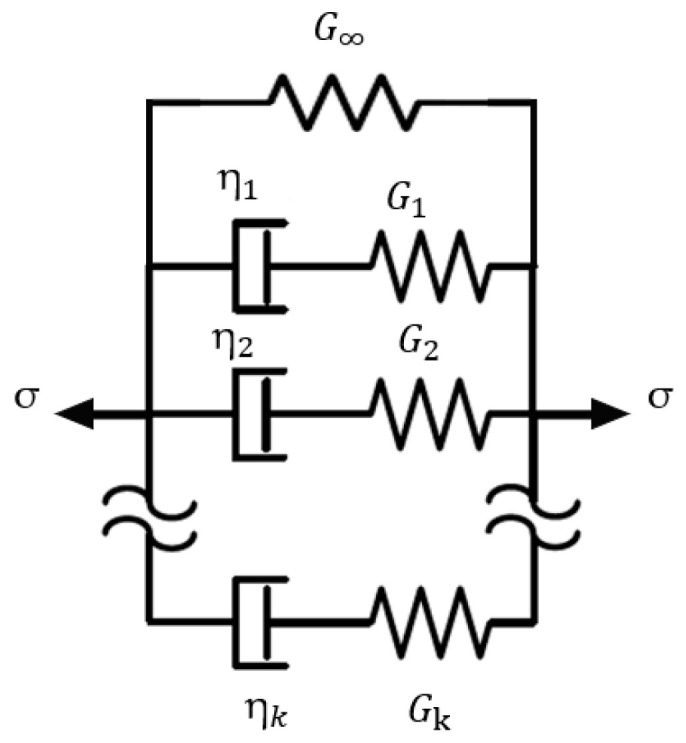
Maxwell–Wiechert model, which employs a combination of springs and dashpots to capture the viscoelastic behaviour of tissue.

Viscoelastic materials possess both viscous (fluid-like) and elastic (solid-like) properties. The response of a viscoelastic material is dependent both on the strain and the strain rate:

(1) $$\sigma =\sigma (\varepsilon ,\;\dot{\varepsilon })$$

where σ is the stress, ε is the strain and $\dot{\varepsilon }$ is the strain rate. It is common to model viscoelastic media as a combination of springs and dashpots for examples the Maxwell–Wiechert model show in Figure 3. The frequency domain response of the system in Figure 3 can be expressed as:

(2) $$G^{*}\left(\omega \right)=G_{\infty }+\sum_{k=1}^{K}G_{k}\frac{\omega^{2}{\tau_{k}}^{2}}{1+\omega^{2}{\tau_{k}}^{2}}+i\sum_{k=1}^{K}G_{k}\frac{\omega \tau_{k}}{1+\omega^{2}{\tau_{k}}^{2}}\;$$

where $G^{*}\left(\omega \right)$ is the complex modulus and ω is the angular frequency, and $\tau_{k}=\eta_{k}/G_{k}$ is the relaxation time [50,51]. For the case of a network with just three components, $G_{\infty }$, $G_{1}$ and $\eta_{1}$, the model reduces to what is referred to the SLS model or Zener model [28,52].

**Figure 4:**
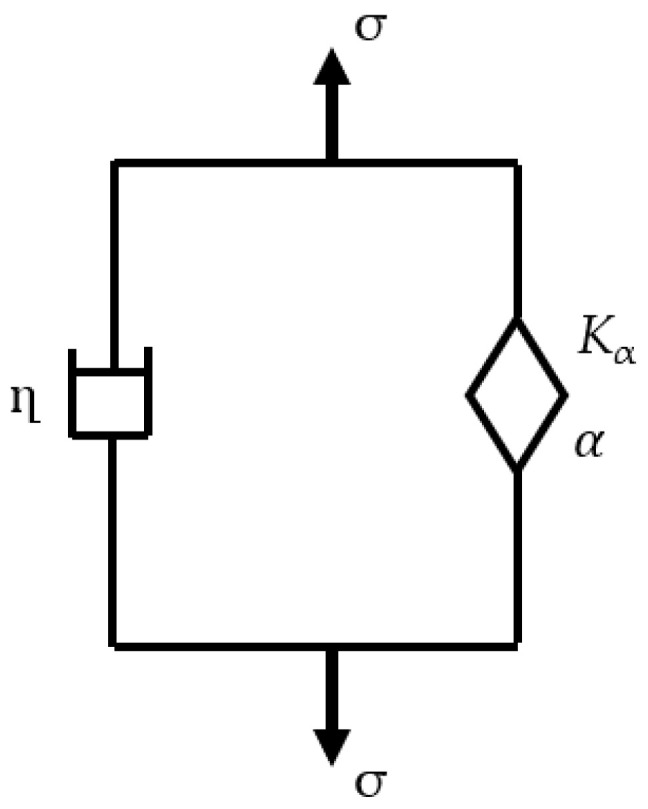
SFKV model used in our study combining a springpot and a dashpot.

An alternative approach for materials that exhibit a power law dependence, including tissues, polymers, gels, emulsions, composites and suspensions, is a fractional derivative model that employs a springpot: a component having intermediate properties between a purely elastic element and a perfectly viscous element [45]. The relationship between its stress (σ) and strain (ε) for a springpot is represented by a fractional order derivative:

(3) $$\sigma =K_{\alpha }\frac{d^{\alpha }\varepsilon }{dt^{\alpha }}$$

where *K*_α_ is the coefficient of consistence (with units of Pa·(s)^α^) and α is the order of fractional derivative (0 ≤ α ≤ 1). The bounding values of α represent the discrete elements employed in conventional viscoelastic models, which is a spring when α = 0 and *K*_α_ = *G* (elastic shear modulus) and a dashpot when α = 1 and *K*_α_ = η (viscosity). The fractional viscoelastic model used in this study uses a springpot in parallel with a dashpot (see Figure 4) referred to as semi-fractional Kelvin–Voigt (SFKV) model, which results in the following stress–strain relationship:

(4) $$\sigma =K_{\alpha }\frac{d^{\alpha }\varepsilon }{dt^{\alpha }}+\eta \frac{d\varepsilon }{dt}$$

In the frequency domain, the storage and loss modulus of the SFKV can be written as [40]:

(5) $$G^{’}\left(\omega \right)=K_{\alpha }\;{\left(\omega \right)}^{\alpha }\;cos\left(\frac{\alpha \pi }{2}\right)$$

(6) $$G^{\prime \prime }\left(\omega \right)=ղ\;\omega +K_{\alpha }\;{\left(\omega \right)}^{\alpha }sin\left(\frac{\alpha \pi }{2}\right)$$

## 3. Results

### 3.1. Strain Sweep

**Figure 5:**
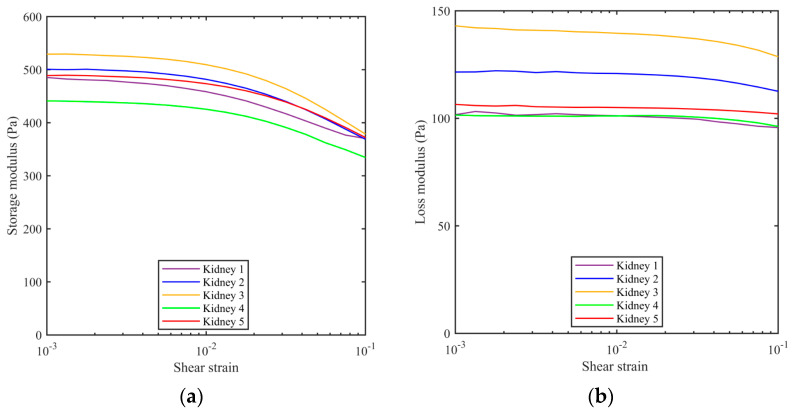
(**a**) Storage modulus and (**b**) loss modulus of porcine kidneys (n = 5) as a function of shear strain at a frequency of 1 Hz. All samples were extracted from the middle pole region of kidneys.

The rheometer was used to determine the storage modulus and loss modulus while increasing the strain amplitude from 0.001 to 0.1 at a frequency of 1 Hz. The dependence of both moduli as a function of strain for five porcine kidneys is depicted in Figure 5. The kidney appears to act as a linear viscoelastic material up to a strain amplitude of 0.01 (the horizontal portions of the curve).

### 3.2. Frequency Sweep

**Figure 6:**
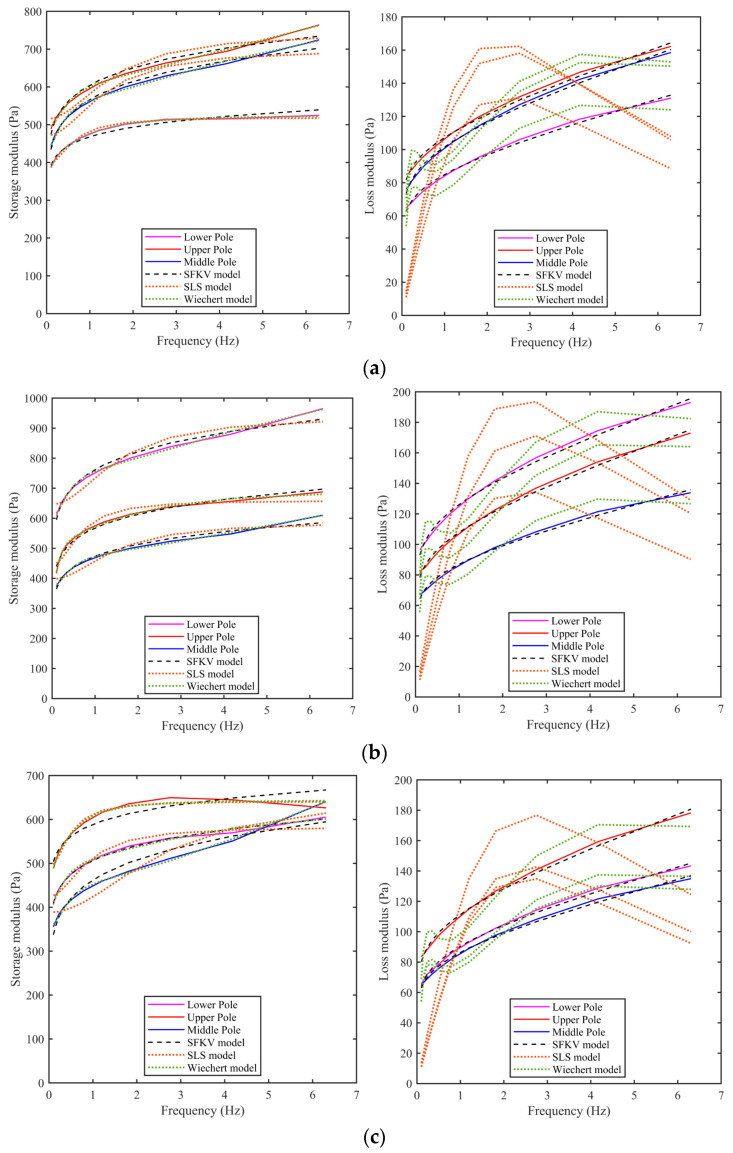
The storage modulus and loss modulus of porcine kidneys 1–3 (**a**–**c**) as a function of frequency along with the model fits. All the measurements were performed at a shear strain of 0.01 with frequency varied from 0.1 Hz to 6.5 Hz.

In the frequency sweep studies, the frequency varied from 0.1 Hz to 6.5 Hz. The upper frequency was where inertial effects in the measurement system were observed to affect the results. The strain amplitude was maintained at 0.01 (i.e., in the linear regime). Figure 6 shows that storage and loss modulus as a function of frequency for the three locations in each of the three kidneys. The experimental data was fit to three models: SFKV, SLS and a five-parameter Maxwell–Wiechert model using MATLAB’s least square fit function. The curves are shown in Figure 6 and the fitted parameters in Table 1, Table 2 and Table 3. It can be seen that all three models match the storage modulus well, but for the loss modulus the SFKV model captures the experimental data best with a correlation coefficient of 0.99. The SLS with a single relaxation time does not capture the frequency domain response well and has a correlation coefficient of 0.53. The five-parameter Maxwell–Wiechert model with the second relaxation time added does significantly better that the SLS, with a correlation coefficient of 0.85.

Table 1 shows the SFKV model parameters extracted from lower pole, upper pole and middle pole of kidneys 1–3 in the frequency domain. There was no significant difference in the SFKV model parameters between different poles and pigs, and the correlation coefficient is 0.99.

Table 2 shows the SLS model parameters extracted from the lower pole, upper pole and middle pole of kidneys 1–3. No significant difference in the SLS model parameters was observed between different poles and pigs. The SLS model well fitted the storage modulus data and was unable to capture the variation in loss modulus with frequency. The correlation coefficient of SLS model is higher than 0.53.

Table 3 shows the values for the five parameter Maxwell–Wiechert model extracted from the lower pole, upper pole and middle pole of kidneys 1–3. There was no significant difference in the Maxwell–Wiechert model parameters observed across different poles and pigs. The Maxwell–Wiechert model well fitted the storage modulus data and was also able to capture the variation in loss modulus with frequency better than the SLS model. The correlation coefficient of Maxwell–Wiechert model is higher than 0.85.

### 3.3. Stress Relaxation

The robustness of the frequency domain fitting was investigated by carrying out a step-shear measurement in the time domain and measuring the relaxation modulus. The measured data was then fitted to both the SLS and fractional models. For the SLS model, the relaxation modulus is given by:

(7) G=G∞+G1 e−tτ; t≥0 

For the fractional viscoelastic model, the relaxation modulus is given by:

(8) $$G\left(t\right)=\eta \;\delta (t)+K_{\alpha }\frac{\;t^{-\alpha }}{Ґ\left(1-\alpha \right)}$$

where δ(t) is the delta function and Ґ is the gamma function.

**Figure 7:**
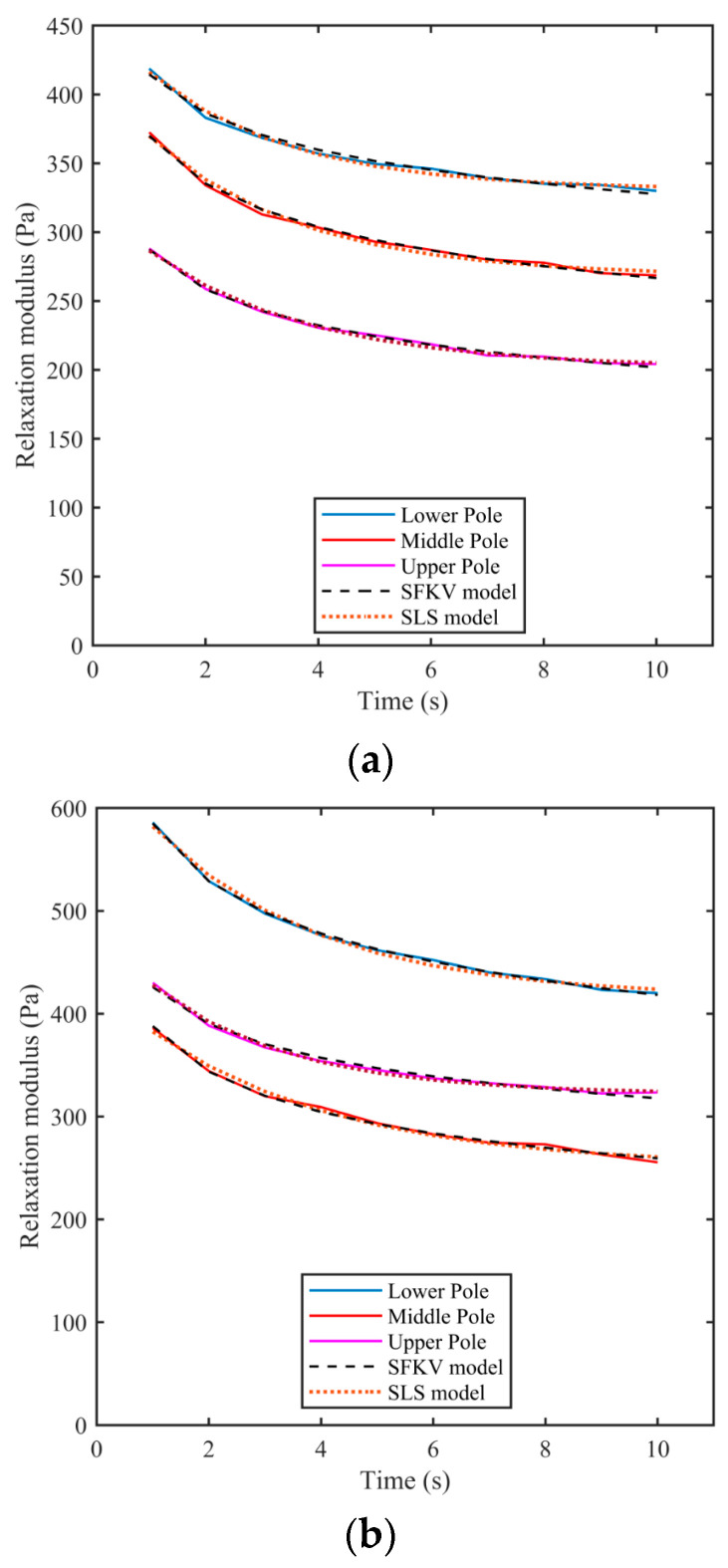

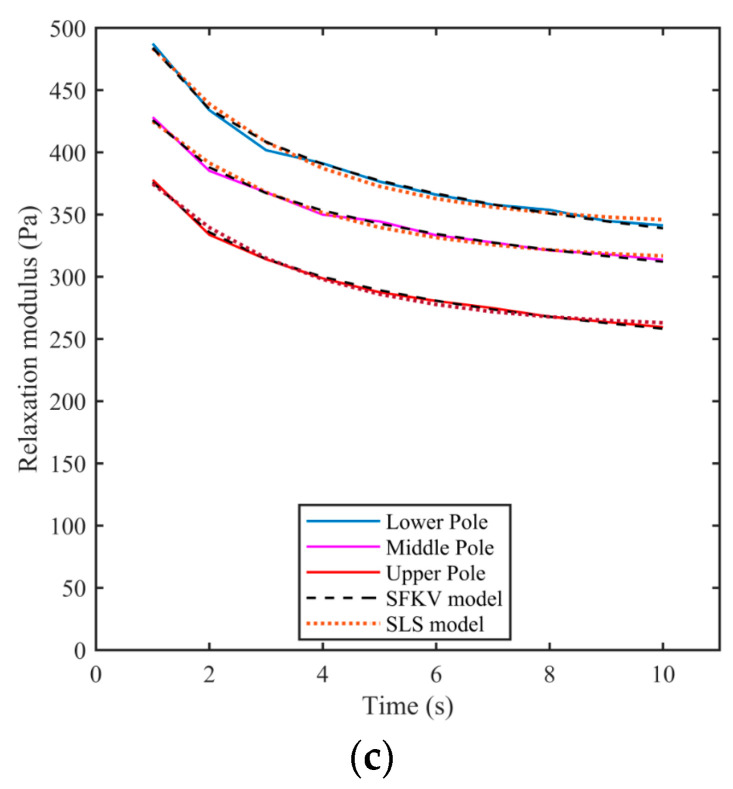
The relaxation modulus of porcine kidneys as a function of time across the lower pole, middle pole and upper pole regions of kidneys 1–3 (**a**–**c**) along with the model fits. All the relaxation tests were performed with samples given a step shear strain of 0.01.

Both the SLS and SFKV models were fitted to the measured relaxation data for all three poles and all three kidneys. Figure 7 shows the measured relaxation modulus as a function of time along with the fitted SLS and SFKV models. Table 4 shows viscoelastic parameters for the SFKV fit. No difference was observed in the relaxation behavior or model parameters across the poles or pigs, and both the SFKV and SLS model fitted with a correlation coefficient of 0.99.

Table 5 shows the Standard Linear Solid (SLS) model parameters extracted from lower pole, upper pole, and middle pole regions of kidney 1–3. There was no significant difference in SLS model parameters across the poles or kidneys, and the correlation coefficient is 0.99.

## 4. Discussion

In this paper, the biomechanical measurements performed on porcine kidneys were reported. It was observed that the kidneys acted as linear viscoelastic materials for shear strains up to 0.01. This is within the range reported by other studies on porcine kidneys, with 0.1 reported in reference [53] and 0.002 reported in reference [1]. Further we found there was a monotonic increase in the storage modulus and loss modulus with frequency for all regions of the kidney. This is consistent with measurements on fresh pig kidneys, which observed a power law between 0.1 and 4 Hz for the storage and loss modulus [53].

In our study, the average storage modulus across the different sites and animals was G′ = 0.48 ± 0.03 kPa and the loss modulus of G″ = 0.12 ± 0.02 kPa at 1 Hz. This compares to: G′ = 1.8 kPa and G″ = 0.2 kPa reported at 0.1 Hz in fresh porcine kidneys using rheometry [53]; G = 2.1 ± 0.35 kPa at 50 Hz to 200 Hz performed using Shear Wave Dispersion Ultrasound Vibrometry measurements on ex vivo porcine kidneys [54]; G = 1.67 kPa at 75 Hz to 300 Hz in ex vivo porcine kidneys using magnetic resonance elastography [55]. A study across different regions of porcine kidneys [56] reported G = 5.2 ± 1 kPa in the cortex region, G = 6.7 ± 1.7 kPa in the medulla region and G = 8.7 ± 2.4 kPa in the pelvis region. The shear modulus obtained in our study varied from a factor of ~4 to ~20 compared to other studies. This is potentially due to the elastography data extracted at frequencies of 100 s of Hz.

Measurements from the kidneys of humans have been reported using ultrasound shear wave elastography with results from the mid pole of the kidney cortex ranging from 9.34 ± 0.99 kPa to 13.05 ± 1.85 kPa [57]; on a finer spatial scale, values were reported to be 4 kPa in glomeruli, 1.5 kPa in tubules and 1 kPa in the interstitium at 100–400 Hz [58]. These data are similar in range to what has been reported in porcine kidneys and support the use of porcine kidneys as a biomechanical model for human kidneys.

In the time domain, a monotonic reduction in the relaxation modulus was observed across all the renal regions with an average relaxation time of 2.7 s. This behavior is similar to the other studies of porcine kidneys with measured relaxation times reported to be ~2 s [1] and ~3.5 s [59]. Therefore, the relaxation time reported in our study is in line with other measurements. 

The constitutive models fitted the frequency and time domain data well, with the exception of the SLS model, which did not capture the frequency dependence of the loss modulus. A five-parameter Maxwell–Wiechert model was able to capture the frequency dependence of both the storage modulus and loss modulus better than the SLS model. This is due to the higher number of parameters that represent the behavior of kidney tissue across a wide range of frequencies. When comparing across the fits, it was observed that for the SFKV model $K_{\alpha }$ was 30% higher for the frequency domain that for the time domain. For the SLS model, the average value of $G_{1}$ was 23% higher in the frequency domain than the time domain, $G_{\infty }$ was 55% higher and η_1_ was 59% lower. This variation is about as large as the differences between the different kidneys and so seems with the variability of the measurements reported here. 

Our data suggest that the SFKV model captures the shear moduli better than the SLS with both models having three parameters. However, implementing the models in simulations does not incur the same computational cost [60]. The advantage of the SLS model is that with only three classical elements it is relatively simple to deploy in simulations using standard differential solvers. On the other hand, the SFKV model only requires a few parameters but in time-stepping computational codes it requires more complex implementation, which can be a barrier to their use [39,61]. We do note that by using additional branches in the SLS model, we have used a Maxwell–Wiechert model to improve the agreement between the model and the data; however, this means more parameters need to be specified and the computational cost increases.

Although significant work has been carried out in the biomechanical modelling of kidneys and other soft tissues, there is a need for improved models to fully understand the complex mechanical behavior of the kidney. The work here used linear isotropic models with just a few parameters. Developing comprehensive models of kidney function and pathology requires integrating data from different scales, such as tissue microstructure, cellular and molecular mechanisms, and organ-level biomechanics. Reference [62] characterized the nonlinear biaxial mechanical properties of human ureter. Reference [63] used a generalised hyperelastic model and decomposed the proposed strain energy functions into an isotropic and an anisotropic part, which corresponded to the histological structure of soft tissues. Reference [64] used the mathematical framework of oblique contravariant tensors and their associated invariants to model damage in anisotropic soft tissues. These models require robust calibration to experimental data, which has significant variability within individual organs, within studies and across studies.

## 5. Conclusions

This research examined the rheological behavior of porcine kidneys in the linear viscoelastic range from frequencies from 0.1 Hz to 6.5 Hz. The SFKV model best fitted the frequency domain data with a correlation coefficient of 0.99. SLS model well fitted the storage modulus in frequency domain and relaxation modulus in time domain but was unable to capture the variation in loss modulus with frequency, and the correlation coefficient was higher than 0.53. A five-element Maxwell–Wiechert model was able to capture the frequency variation in storage modulus and loss modulus better than the SLS model with a correlation coefficient higher than 0.85. Both SLS model and SFKV model fitted the time-domain data with a correlation coefficient of 0.99. The SFKV, SLS and Maxwell–Wiechert models will be useful in computational modelling of the stress–strain response of kidneys for a variety of mechanical loads.

## Figures and Tables

**Table 1:** Values of SFKV model parameters extracted from kidney 1, 2 and 3.

Kidney	Pole	*K*_α_ [Pa·(s)^α^]	α	η [Pa·s]
Kidney 1	Lower	473	0.09	10.46
	Upper	613	0.11	6.45
	Middle	576	0.11	5.19
Kidney 2	Lower	773	0.10	6.17
	Upper	577	0.11	8.27
	Middle	482	0.11	4.75
Kidney 3	Lower	513	0.10	9.07
	Upper	595	0.08	16.99
	Middle	472	0.13	1.61
Average		564 ± 96	0.10 ± 0.01	7.66 ± 4.36

**Table 2:** Values of SLS model parameters extracted from kidney 1, 2 and 3.

Kidney	Pole	$G_{1}$ (Pa)	$G_{\infty }$ (Pa)	$\eta_{1}$ (Pa·s)
Kidney 1	Lower	125	393	199
	Upper	227	515	146
	Middle	227	471	167
Kidney 2	Lower	291	647	190
	Upper	213	446	313
	Middle	190	398	129
Kidney 3	Lower	158	425	177
	Upper	150	491	311
	Middle	263	388	103
Average		205 ± 54	464 ± 82	193 ± 74

**Table 3:** Values of a five-element Maxwell–Wiechert model parameters extracted from kidney 1, 2 and 3.

Kidney	Pole	$G_{1}$ (Pa)	$G_{\infty }$ (Pa)	$\eta_{1}$ (Pa·s)	$G_{2}$ (Pa)	$\eta_{2}$ (Pa·s)
Kidney 1	Lower	68	370	368	86	79
	Upper	131	491	264	356	46
	Middle	135	438	358	258	48
Kidney 2	Lower	163	607	400	365	62
	Upper	170	378	960	146	72
	Middle	114	373	262	338	41
Kidney 3	Lower	110	404	313	117	32
	Upper	79	447	772	119	185
	Middle	109	355	249	614	62
Average		120 ± 34	429 ± 80	438 ± 252	266 ± 171	70 ± 46

**Table 4:** Values of SFKV model parameters fit to the time domain data from kidney 1, 2 and 3.

Kidney	Pole	*K*_α_ [Pa·(s)^α^]	α
Kidney 1	Lower	443	0.10
	Middle	409	0.14
	Upper	321	0.15
Kidney 2	Lower	648	0.15
	Middle	441	0.18
	Upper	465	0.13
Kidney 3	Lower	541	0.16
	Middle	468	0.14
	Upper	423	0.16
Average		462 ± 91	0.15 ± 0.02

**Table 5:** Values of Standard Linear Solid (SLS) model parameters fit to the time domain data from kidney 1, 2 and 3. The equivalent viscosity $\eta_{1}$ =$\;\tau_{1}\times G_{1}$ for the average relaxation time is 466 Pa.

Kidney	Pole	$G_{1}$ (Pa)	$G_{\infty }$ (Pa)	τ (s)
Kidney 1	Lower	128	331	2.49
	Middle	148	268	2.69
	Upper	121	202	2.84
Kidney 2	Lower	233	416	2.98
	Middle	176	251	3.42
	Upper	160	322	2.44
Kidney 3	Lower	208	341	2.66
	Middle	160	312	2.85
	Upper	167	259	2.78
Average		167 ± 36	300 ± 63	2.79 ± 0.29

## Data Availability

The data will be made available by the authors on request.
